# Characterisation of CCT271850, a selective, oral and potent MPS1 inhibitor, used to directly measure *in vivo* MPS1 inhibition *vs* therapeutic efficacy

**DOI:** 10.1038/bjc.2017.75

**Published:** 2017-03-23

**Authors:** Amir Faisal, Grace W Y Mak, Mark D Gurden, Cristina P R Xavier, Simon J Anderhub, Paolo Innocenti, Isaac M Westwood, Sébastien Naud, Angela Hayes, Gary Box, Melanie R Valenti, Alexis K De Haven Brandon, Lisa O'Fee, Jessica Schmitt, Hannah L Woodward, Rosemary Burke, Rob L M vanMontfort, Julian Blagg, Florence I Raynaud, Suzanne A Eccles, Swen Hoelder, Spiros Linardopoulos

**Affiliations:** 1 Cancer Research UK Cancer Therapeutics Unit, Division of Cancer Therapeutics, The Institute of Cancer Research, London, UK; 2 Breast Cancer Now, Division of Breast Cancer Research, The Institute of Cancer Research, London, UK

**Keywords:** MPS1, inhibition, spindle assembly checkpoint, pharmacodynamics

## Abstract

**Background:**

The main role of the cell cycle is to enable error-free DNA replication, chromosome segregation and cytokinesis. One of the best characterised checkpoint pathways is the spindle assembly checkpoint, which prevents anaphase onset until the appropriate attachment and tension across kinetochores is achieved. MPS1 kinase activity is essential for the activation of the spindle assembly checkpoint and has been shown to be deregulated in human tumours with chromosomal instability and aneuploidy. Therefore, MPS1 inhibition represents an attractive strategy to target cancers.

**Methods:**

To evaluate CCT271850 cellular potency, two specific antibodies that recognise the activation sites of MPS1 were used and its antiproliferative activity was determined in 91 human cancer cell lines. DLD1 cells with induced GFP-MPS1 and HCT116 cells were used in *in vivo* studies to directly measure MPS1 inhibition and efficacy of CCT271850 treatment.

**Results:**

CCT271850 selectively and potently inhibits MPS1 kinase activity in biochemical and cellular assays and in *in vivo* models. Mechanistically, tumour cells treated with CCT271850 acquire aberrant numbers of chromosomes and the majority of cells divide their chromosomes without proper alignment because of abrogation of the mitotic checkpoint, leading to cell death. We demonstrated a moderate level of efficacy of CCT271850 as a single agent in a human colorectal carcinoma xenograft model.

**Conclusions:**

CCT271850 is a potent, selective and orally bioavailable MPS1 kinase inhibitor. On the basis of *in vivo* pharmacodynamic *vs* efficacy relationships, we predict that more than 80% inhibition of MPS1 activity for at least 24 h is required to achieve tumour stasis or regression by CCT271850.

Dividing cells faithfully transmit their genetic information into daughter cells by accurately segregating duplicated chromosomes during mitosis. This is ensured by a surveillance mechanism called the spindle assembly checkpoint (SAC, also known as mitotic checkpoint), which delays mitotic progression until all chromosomes are properly attached to spindle microtubules (Musacchio and Salmon, 2007; Lara-Gonzalez *et al*, 2012; Foley and Kapoor, 2013). Inactivation of SAC results in premature anaphase onset and, therefore, missegregation of erroneously attached chromosomes. This consequently leads to chromosomal instability and aneuploidy that can either cause cell death or tumorigenesis depending on the strength of checkpoint abrogation (Dobles *et al*, 2000; Kops *et al*, 2004, 2005; Gordon *et al*, 2012). The dual specificity kinase, MPS1 (also known as TTK), is one of the core proteins that regulates SAC activation by initiating and transducing an inhibitory signal from unattached kinetochores to anaphase-promoting complex/cyclosome (APC/C), an E3 ubiquitin ligase (Musacchio and Salmon, 2007). Consequently, APC/C-mediated degradation of the mitotic proteins cyclin B and securin, normally required for mitotic exit, is inhibited and cells remain arrested in mitosis until all chromosomes are properly attached (Lara-Gonzalez *et al*, 2012). Incidentally, MPS1 kinase activity can also regulate chromosomal attachment and error correction (Maure *et al*, 2007; Jelluma *et al*, 2008). Loss of MPS1 activity, therefore, results in both abrogation of SAC function and chromosomal misalignment, ultimately leading to severe aneuploidy and apoptotic cell death (Schmidt *et al*, 2005; Jelluma *et al*, 2008; Hewitt *et al*, 2010; Kwiatkowski *et al*, 2010; Santaguida *et al*, 2010).

A large majority of human cancers are characterised by aneuploidy and chromosomal instability, despite normal cells being highly intolerant to both phenotypes (Orr *et al*, 2015). Overexpression of several SAC components, including MPS1 kinase, have been suggested as one of the mechanisms through which tumour cells can tolerate high aneuploidy and chromosomal instability (Yuan *et al*, 2006; Daniel *et al*, 2011). MPS1 is overexpressed in various human tumours, including breast, pancreatic, thyroid and glioblastoma, and its higher expression levels correlate with poor prognosis in many of these tumours (Salvatore *et al*, 2007; Daniel *et al*, 2011; Tannous *et al*, 2013; Slee *et al*, 2014). Reduction in MPS1 levels or activity in such tumours can result in loss of cell viability (Daniel *et al*, 2011; Slee *et al*, 2014), or in increased sensitivity to low doses of microtubule-polymerising agents such as docetaxel (Tannous *et al*, 2013; Maia *et al*, 2015). Therefore, MPS1 inhibition represents an attractive strategy to target cancers, especially those with chromosomal instability (Manchado *et al*, 2012). Several structurally diverse MPS1 inhibitors have been discovered and have undergone preclinical assessments in recent times (Liu and Winey, 2012). These include AZ3146 (Hewitt *et al*, 2010), NMS-P715 (Colombo *et al*, 2010), MPI-0479605 (Tardif *et al*, 2011), CCT251455 (Naud *et al*, 2013), MPS1-IN-3 (Tannous *et al*, 2013) and compounds from Shionogi (Kusakabe *et al*, 2015) and Bayer (Jemaa *et al*, 2013).

The availability of robust pharmacodynamic (PD) biomarkers is critical for any modern drug development programme (Workman, 2003). Inhibition of histone H3 phosphorylation at S10, or downregulation of mitotic proteins such as cyclin B, have been widely used as biomarkers for SAC over-ride and the accompanying mitotic exit that results from cellular inhibition of MPS1 (Colombo *et al*, 2010; Kwiatkowski *et al*, 2010; Tardif *et al*, 2011; Naud *et al*, 2013). Of several MPS1 inhibitors that have been reported, only a few have demonstrated direct MPS1 inhibition in cells using either phospho-specific antibodies for the MPS1 T676 autophosphorylation site, or a change in mobility of MPS1 by SDS–PAGE (Colombo *et al*, 2010; Hewitt *et al*, 2010; Kwiatkowski *et al*, 2010; Tardif *et al*, 2011). For many inhibitors that have been evaluated in *in vivo* models, although not exclusively, histone H3 phosphorylation at S10 has been the biomarker of choice to demonstrate MPS1 inhibition in tumours (Colombo *et al*, 2010; Jemaa *et al*, 2013). SAC over-ride and consequential reduction in histone H3 phosphorylation at S10 are, however, not specific for MPS1 inhibition and can be regulated by other kinases involved in the SAC (Mao *et al*, 2003; Santaguida *et al*, 2011; Saurin *et al*, 2011).

Our efforts to discover MPS1 inhibitors with favourable biochemical, cellular, pharmacokinetic and PD and *in vivo* properties for a clinical candidate have yielded inhibitors from two different chemical series: CCT251455 (Naud *et al*, 2013) and CCT271850 (Innocenti *et al*, 2016). Here we report an extensive cellular characterisation and *in vivo* parmacodynamic *vs* efficacy relationship of CCT271850.

## Materials and methods

### Cell culture, transfection and proliferation assay

Cell lines were obtained from the American Type Culture Collection and grown in their recommended culture medium, supplemented with 10% FBS at 37 °C in 5% CO_2_. In-house authentication of cell lines by SNP profiling was carried out and cultured cells were passaged for less than 6 months before replacement from early-passage frozen stocks. Cells were regularly screened for Mycoplasma, using a PCR-based assay (VenorGem; Minerva Biolabs, Berlin, Germany). Transfections were carried out at ∼80% confluency with the plasmids indicated, using Lipofectamine LTX (15338030, Life Technologies, Carlsbad, CA, USA) according to the manufacturer's instructions. Cell proliferation assays were carried out by colorimetric MTT method (Sigma, St Louis, MO, USA) as described elsewhere (Mosmann, 1983). Briefly, cells were plated in 96-well plates at 2000–5000 cells per well (depending on the cell line) followed by treatment with two-fold dilutions of 0–25 *μ*M drug for 72 h. Absorbance was measured at 570 nm with the Wallac VICTOR2 1420 Multilabel Counter (PerkinElmer, Waltham, MA, USA).

### Immunoprecipitation and immunoblotting

For immunoprecipitation assay, 1 mg of total cell/tumour lysate was incubated with 8 *μ*g of pTpS^33/37^ antibody (44–1325 G, Life Technologies) for 1 h with rotation at 4 °C. The antibody/protein complexes were captured by further incubation with Protein A sepharose beads for 1 h with rotation at 4 °C. Beads were washed three times with completed lysis buffer, followed by boiling for 10 min in 2 × LDS sample buffer. The samples were used for immunoblotting with total MPS1 antibodies as described below.

For immunoblotting, cells were lysed either in NP-40 alternative (Calbiochem, Merck Millipore, Billerica, MA, USA) lysis buffer (120 mmol/l NaCl, 50 mmol/l Tris-HCl, pH 7.5, 1% NP-40 supplemented with phosphatase and protease inhibitors) or 2 × LDS sample buffer (Invitrogen, Carlsbad, CA, USA). Equal amounts of proteins were resolved by 4–12% Bis-Tris NuPAGE gels (Invitrogen), transferred to nitrocellulose (Whatman, Buckinghamshire, UK) membranes and immunoblotted with specific antibodies. For gel shift analysis, samples were prepared in SDS-sample buffer and resolved by 10% SDS–PAGE.

### Immunofluorescence and time-lapse microscopy

For immunofluorescence, cells were pretreated with CCT271850 for 1 h, then treated with nocodazole, MG132 and CCT271850 for additional 1 h. Cells were fixed and stained according to the protocol previously described (Gurden *et al*, 2015). Primary antibodies used were as follows: anticentromere antibodies (ACA; ImmunoVision, Springdale, AR, USA, HCT-0100), BubR1 (BD Biosciences, San Jose, CA, USA, 612503), Mad1 (Abcam, Cambridge, UK, ab45286), Mad2 (Bethyl Laboratories Inc., Montgomery, TX, USA, A300-301A), MPS1 (Invitrogen, 35–9100), MPS1 pT33pS37 (Life Technologies, 44–1325 G) and Zwint-1 (Abcam, ab84367). Images were acquired using a Zeiss LSM 710 confocal microscope and processed using the Volocity 3D Image analysis software (PerkinElmer). Time-lapse microscopy was performed in 96-well Ibidi plate (Thistle Scientific, Glasgow, UK) using a Diaphot inverted microscope (Nikon, Tokyo, Japan), in a humidified CO_2_ chamber at 37 °C, using a motorised stage (Prior Scientific, Cambridge, UK), controlled by Simple PCI software (Compix, Irvine, CA, USA).

### Flow cytometry

After treatment with CCT271850 for 24 h, cells were fixed and stained as described previously (Gurden *et al*, 2015). Briefly, cells were fixed in 70% ethanol, washed in PBS, incubated in 10 *μ*g/ml propidium iodide and 0.5% RNase (Sigma) for 30 min and analysed using LSRII flow cytometer (BD Biosciences). To stain for mitosis, cells were incubated for 1 h at 4 °C with anti-MPM2 antibodies (Millipore, Billerica, MA, USA, 05–368), followed by 1-h incubation at 4 °C with FITC-conjugated secondary antibodies (Life Technologies).

### MSD assay

MSD assay for detection of autophosphorylation of MPS1 at pT33pS37 sites in cell lysates has previously been described (Naud *et al*, 2013). For MSD from tumours, samples were prepared in complete lysis buffer (50 mM NaCl, 20 mM Tris pH 7.5, 1 mM EDTA, 1 mM EGTA, 1% (*v/v*) Triton X-100, 10 mM NaF, protease inhibitor tablet and phosphatase inhibitor cocktails) and 25–37.5 *μ*g protein per well was used for the detection of phosphorylation at T33/S37. We also measured total MPS1 levels in these tumour samples with an MSD assay for GFP, which was developed using mouse monoclonal MPS1 antibody (Invitrogen, Cat no. 35–9100) as capture antibody and rabbit polyclonal GFP antibody (Abcam, Cat no. ab290 at 1 : 2000 dilution) as the detection antibody. Results were presented as the ratio of Phospho-MPS1/Total MPS1.

### *In vivo* mouse PK/PD study

Overall, 5 × 10^6^ of MPS1-doxycycline (Dox)-inducible DLD-1 human colorectal carcinoma cells were injected s.c. bilaterally into the flanks. Once tumours reached a mean diameter of 8–10 mm, animals were put on Dox diet for 3 days and given an oral gavage bolus of Dox (6 mg/mouse) 24 h before dosing of compounds. Animals (*n*=3 per group) were dosed once daily with CCT271850 (50 or 100 mg/kg po) or vehicle (10% DMSO, 5% Tween 20, 85% saline). Mice were culled at 2, 6, 12 and 24 h after dosing. Heparinised plasma was collected and tumours were snap-frozen for pharmacokinetics (PK) and PD biomarker analysis. For PK examination, compounds were extracted from whole blood, plasma and tissue homogenates with methanol-containing internal standards using established protocols. CCT271850 concentrations were determined using liquid chromatography/tandem mass spectrometry and PK were calculated using the Pharsight Phoenix Software (Mountain View, CA, USA, version 6.3). For PD examination, samples were lysed and analysed by MSD for pT33pS37and GFP levels and ratio of P-MPS1 (pT33pS37)/Total-MPS1 (GFP) in tumour samples at various time points after 50 and 100 mg/kg dosing were calculated.

### Efficacy study

Three million HCT116 human colorectal carcinoma cells were injected s.c. into the right flanks of athymic mice. Therapy was initiated when tumours reached a mean diameter of 5 mm (indicated as day 0). Control mice (*n*=7) received vehicle (10% DMSO, 5% Tween 20, 85% saline) and treated mice (*n*=8) were given CCT271850 at 50 and 100 mg/kg orally twice daily with a break after 1 week's dosing. Tumour volumes and body weights were measured three times weekly and the study was terminated on day 15 when all tumours were excised and weighed. Plasma and tumour samples were collected for pharmacokinetic and PD biomarker analysis at 2 and 6 h after the final dose.

All animal studies were approved by the local research ethics committee and carried out in accordance with the UK Animals (Scientific Procedures) Act 1986 and national guidelines (Workman *et al*, 2010).

## Results

### CCT271850 potently inhibits MPS1 phosphorylation and activity in cells

**Figure 1:**
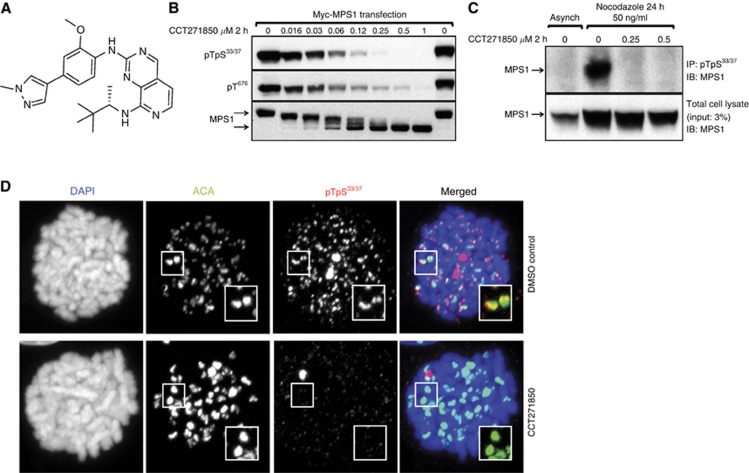
**CCT271850 inhibits MPS1 autophosphorylation in cells.**(**A**) Structure of CCT271850. (**B**) HCT116 cells transiently expressing Myc-tagged MPS1 were treated with increasing concentrations of CCT271850 for 2 h. Cell lysates were analysed for MPS1 autophosphorylation (at pT33pS37 and pT676 sites) and gel shift using immunoblotting. (**C**) HCT116 cells were synchronised with nocodazole for 24 h followed by treatment with indicated concentrations of CCT271850 for 2 h in the presence of MG132. Cell lysates were immunoprecipitated for endogenous MPS1 with phospho-specific anti-pT33pS37 antibodies and immunoblotted with anti-MPS1 antibodies. Total cell lysates were also immunoblotted with anti-MPS1 antibodies as a control for immunoprecipitation input (lower panel). (**D**) Immunofluorescence for MPS1 pT33pS37 localisation at kinetochores with and without treatment with CCT271850. Cells were treated with CCT271850 for 1 h, followed by treatment with nocodazole, MG132 and CCT271850 for an additional hour. Cells were fixed and stained as indicated.

CCT271850 (Figure 1A) is a novel and potent small molecule inhibitor of MPS1 kinase discovered through our lead optimisation studies on a pyridopyrimidine series of compounds (Innocenti *et al*, 2016; compound 34 h). In an *in vitro* kinase assay, CCT271850 inhibited MPS1 activity with IC_50_ values of 0.0112±0.0046 *μ*M at low ATP (10 *μ*M) and 0.02±0.013 *μ*M at high ATP (1 mM) concentrations (Table 1). For cellular inhibition of MPS1, we evaluated the effect of the inhibitor on MPS1 autophosphorylation at the activation site T676, and the N-terminal sites T33/S37 in human colon cancer HCT116 cells (Figure 1B). CCT271850 potently inhibited phosphorylation of ectopically expressed MPS1 at both sites in a dose-dependent manner (Figure 1B). The decrease in phosphorylation was accompanied by corresponding increase in mobility of total MPS1 in SDS–PAGE – another measure of the decrease in MPS1 phosphorylation (Figure 1B, lower panel).

The N-terminal phosphorylation sites of the MPS1 protein have previously been identified as autophosphorylation sites through mass spectrometry of *in vitro* as well as *in vivo* phosphorylated MPS1 protein (Jelluma *et al*, 2008; Xu *et al*, 2009). Here we used phospho-MPS1 pThr33/pSer37 antibody (pT33pS37) to demonstrate recombinant MPS1 is indeed autophosphorylated at these sites in an *in vitro* kinase assay (Supplementary Figure S1A). This autophosphorylation was previously suggested to be inhibited by the MPS1 inhibitor AZ3146; however, it has not been demonstrated (Hewitt *et al*, 2010). The lack of phosphorylation on MPS1 T33A and S37A mutants confirmed the specificity of the antibodies (Supplementary Figure S1C). We were also able to detect phosphorylation of the endogenous MPS1 T33S37 in immunoprecipitation assays following nocodazole-mediated mitotic arrest of HCT116 cells and treatment with CCT271850 in the presence of the proteasome inhibitor MG132 to block mitotic abrogation (Figure 1C), as well as when using the previously reported MPS1 inhibitor, CCT251455 (Naud *et al*, 2013; Supplementary Figure S1B). MPS1 phosphorylation was strongly induced upon nocodazole-mediated mitotic arrest and was completely inhibited by treatment of arrested cells with CCT271850. In addition, phosphorylation of the endogenous MPS1 at T33/S37 sites in mitotic cells and its inhibition by CCT271850 were demonstrated by immunofluorescence using pT33pS37 antibodies (Figure 1D). pT33pS37 staining was co-localized with ACA at kinetochores, but was completely lost upon treatment with CCT271850. To quantitatively measure MPS1 autophosphorylation in cells at the T33/S37 sites, we optimised an electrochemiluminescence (Meso Scale Discovery, MSD) assay (Naud *et al*, 2013). Using this approach we showed that CCT271850 potently inhibited MPS1 T33/S37 phosphorylation with IC_50_ value of 0.059±0.022 *μ*M (Table 1). Furthermore, we showed that treatment of nocodazole-arrested HCT116 cells with CCT271850 caused SAC abrogation as measured by inhibition of the histone H3 phosphorylation at S10 with an IC_50_ value of 0.067±0.004 *μ*M (Table 1). Moreover, when compared side-by-side with NMS-P715, CCT271850 was approximately eight-fold more potent (Supplementary Figure S1D).

### Effects of CCT271850 on the activation of SAC and the cell cycle

**Figure 2:**
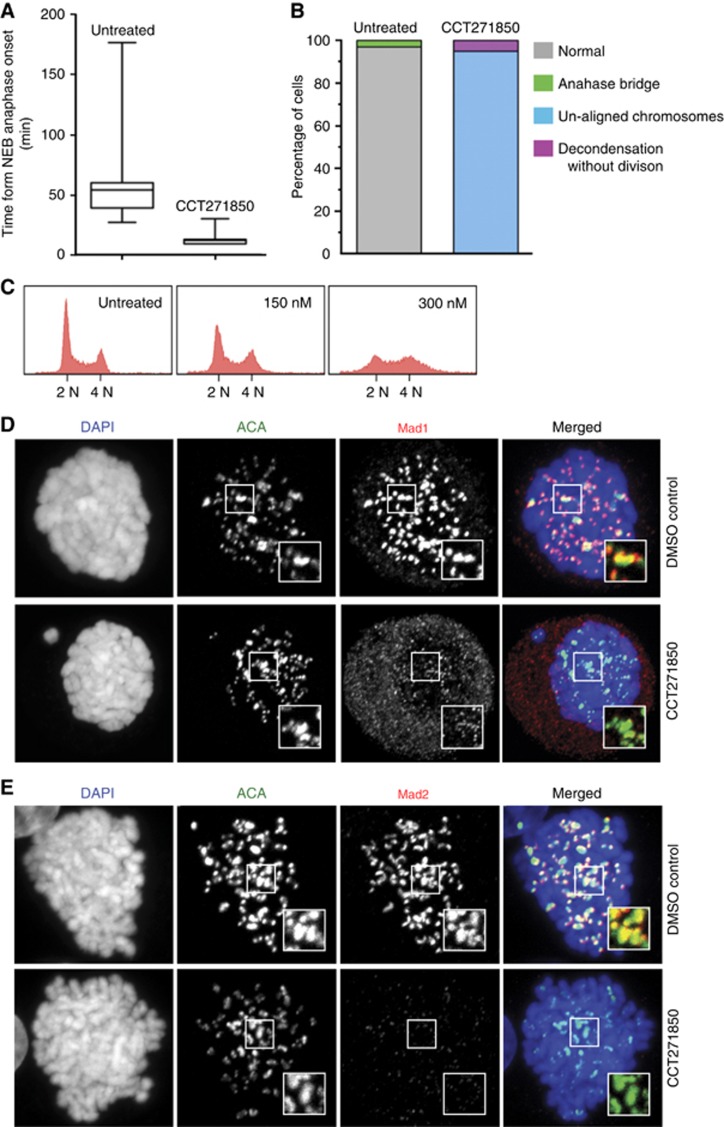
**Mitotic defects in cells treated with CCT271850.**(**A**) Time from nuclear envelope breakdown to anaphase onset in asynchronous HeLa cells stably expressing Histone H2B-mCherry was measured using time-lapse microscopy in the presence or absence of 0.3 *μ*M of CCT271850. Images were taken every 3 min for 24 h. (**B**) Quantification of mitotic defects of HeLa cells from **A**. (**C**) Cell cycle analysis of HCT116 cells treated with the indicated concentrations of CCT271850 for 24 h. (**D**, **E**) Immunofluorescence for Mad1 and Mad2 localisation at kinetochores with and without treatment with CCT271850. Cells were treated with CCT271850 for 1 h, followed by treatment with nocodazole, MG132 and CCT271850 for an additional hour. Cells were fixed and stained as indicated.

The SAC ensures accurate chromosomal segregation by delaying the onset of anaphase until all chromosomes are properly attached to the spindle poles (Lara-Gonzalez *et al*, 2012). MPS1 activity is required for activation of the SAC (Kang *et al*, 2007) and inhibition of its activity results in SAC over-ride and missegregation of chromosomes (Colombo *et al*, 2010; Tardif *et al*, 2011). HeLa cells treated with CCT271850 showed an early exit from mitosis (Figure 2A). Cells treated with 0.3 *μ*M of CCT271850 spent, on average, 12 min to pass from nuclear envelope breakdown to the onset of anaphase, compared to 57 min for the untreated control cells (Figure 2A). The observed SAC abrogation resulted in a large percentage of cells undergoing cell division with unaligned chromosomes, resulting in aneuploidy and a loss of cell cycle profiles by flow cytometry (Figure 2B and C). It is known that MPS1 activity is required for recruitment of spindle checkpoint proteins to kinetochores (London and Biggins, 2014). We therefore tested the recruitment of Mad1, Mad2 and BubR1 to the unattached kinetochores (Hewitt *et al*, 2010; Sliedrecht *et al*, 2010) after treatment of HeLa cells with CCT271850. The results showed that Mad1, Mad2 and BubR1 levels were abolished at the unattached kinetochores (Figure 2D and E and Supplementary Figure S2A), whereas Zwint-1 levels remain unchanged (Supplementary Figure S2B).

### MPS1 inhibition by CCT271850 sensitises selectively MSI+ colon cancer and basal breast cancer cell lines to cell death

**Figure 3:**
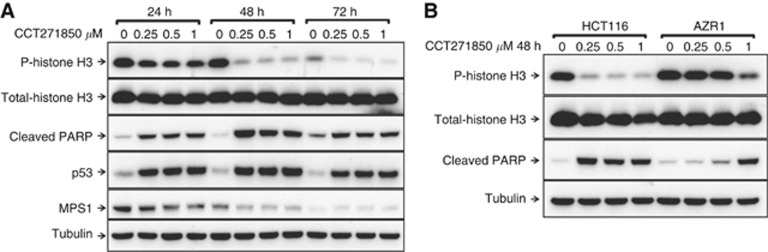
**Biomarker modulation and antiproliferative effects of CCT271850.**(**A**) Time course of biomarker modulation by CCT271850 in HCT116 cells. Cells were treated with indicated concentrations of the inhibitor for 24, 48 and 72 h. Cell lysates were analysed by immunoblotting for inhibition of histone H3 phosphorylation at S10, induction of p53 and PARP cleavage. Total histone H3 and alpha-tubulin were used as loading controls. (**B**) Biomarker modulation by CCT271850 is reversed in AZR1 cell line expressing an inhibitor-resistant mutant of MPS1. HCT116 WT and AZR1-resistant cell lines were treated with indicated concentrations of CCT271850 for 48 h and analysed for inhibition of histone H3 and PARP cleavage as described above.

We further investigated the cellular effect of CCT271850 on asynchronous HCT116 cells. Cells were treated with different concentrations of CCT271850 for 24, 48 and 72 h and inhibition of histone H3 phosphorylation and PARP cleavage was examined by immunoblotting. As shown in Figure 3A, histone H3 phosphorylation at S10 was increasingly inhibited in a time-dependent manner by all three concentrations of the compound. Induction of apoptotic cell death upon CCT271850 treatment also increased in a time-dependent manner as determined by the levels of cleaved PARP. A marked increase on p53 levels in HCT116 cells upon treatment with CCT271850 for 24 and 48 h is consistent with induced p53 response due to aneuploidy (Tardif *et al*, 2011). Treatment with CCT271850 for 24 and 48 h also resulted in a reduction of MPS1 protein levels, indicating mitotic exit (Cui *et al*, 2010).

We have recently generated HCT116 and DLD1 cell lines resistant to a variety of MPS1 inhibitors due to specific point mutations in MPS1 kinase domain (Gurden *et al*, 2015). To determine whether the cellular effects seen upon CCT271850 treatment in Figure 3A were selectively caused by MPS1 inhibition (Figure 3A), we used the AZR1-resistant HCT116 cells carrying the MPS1 S611G mutation, as a model. As shown in Figure 3B, inhibition of histone H3 phosphorylation by CCT271850 in AZR1 cells was abolished compared with the parental HCT116 cells. Similarly, PARP cleavage upon CCT271850 treatment was significantly reduced in AZR1 cell line, indicating that the apoptotic cell death induced by CCT271850 is mediated by MPS1 inhibition. Inhibition of P-histone H3 and PARP cleavage by CCT271850 in AZR1 cell lines was seen only at higher concentrations of CCT271850 (⩾1 *μ*M; Figure 3B and Supplementary Figure S3).

To evaluate CCT271850 antiproliferation effects in a large panel of human cancer cell lines, 91 cell lines were treated for 72 h and 14 days at different compound concentrations in growth inhibition assays. The growth inhibition values as measured by IC_50_ are summarised in Table 2 and Supplementary Table S1. In summary, at 72 h of CCT271850 treatment, more than half the cell lines (57/91) showed IC_50_ values below the average of 1.588 *μ*M, and nearly half (45/91) showed an IC_50_ at submicromolar values. Cell lines from colon and head and neck cancers were among the most sensitive. Importantly, microsatellite instability-positive (MSI+) colon cancer cell lines were more sensitive to cell death by CCT271850 in comparison to microsatellite stable (MSS) cell lines (average IC_50_ at 72 h: MSI+=0.276±0.06, MSS=1.31±0.06; *P*=0.03; Table 2). In addition, in a panel of 20 breast cancer cell lines, we found that the basal, as well as specifically the PTEN-deficient basal cancer cells, were more sensitive to cell death by CCT271850 in comparison to luminal breast cancer cell lines (average IC_50_ at 96 h: Basal=0.7±0.2, Luminal=2.1±0.54; *P*=0.014; Table 2).

### Development of PD biomarker assay for measuring direct inhibition of MPS1 in xenograft tumours

**Figure 4:**
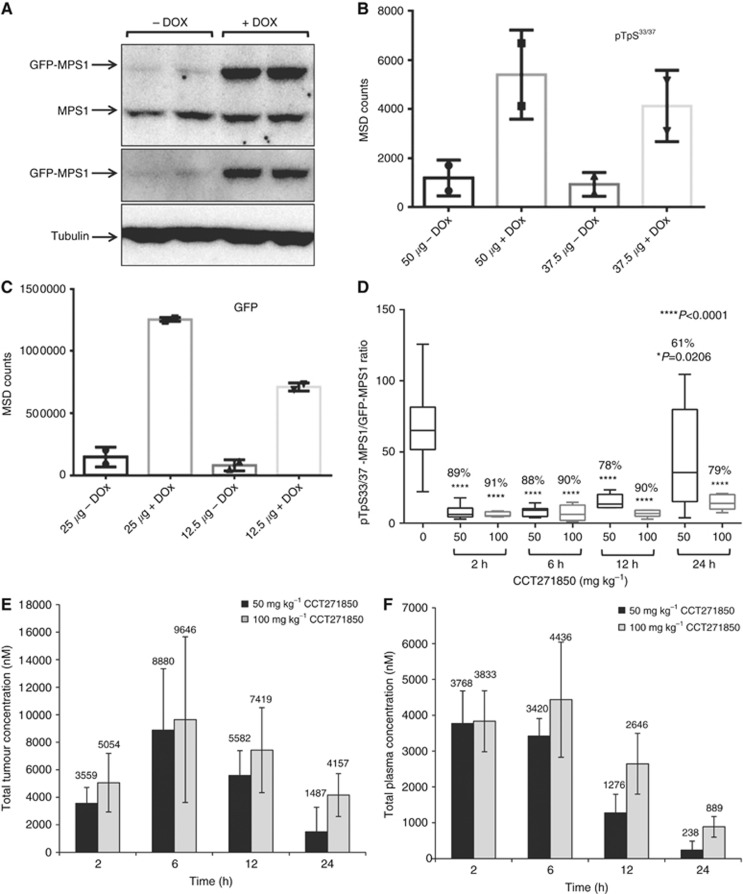
**Development of a PD biomarker assay for measuring direct inhibition of MPS1 in DLD1 GFP-MPS1 Dox-inducible xenografts.**(**A**) Five million DLD1 cells, stably expressing Dox-inducible GFP-MPS1, were used to grow tumours in athymic mice. Mice were dosed with ∼6 mg of Dox per day for 3 days. Tumours were lysed and equal amount of protein was used for immunoblotting with GFP and MPS1 antibodies. (**B**) MSD assay for detection of MPS1 phosphorylation in xenograft tumours. Cell lysates (37.5 and 50 *μ*g) from non-induced and induced tumours were used for MSD assay with pTpS^33/37^ antibodies. (**C**) Two concentrations (12.5 and 25 *μ*g) of cell lysates were also used for MSD with GFP antibodies. (**D**) PK/PD studies in DLD1 GFP-MPS1 Dox-inducible xenografts treated with CCT271850. Mice bearing bilateral DLD1 (GFP-MPS1 Dox inducible) xenografts were placed on Dox diet for 3 days (∼6 mg/day). Twenty-four hours prior to harvest, mice were given a single 10 mg oral gavage bolus of Dox, followed by a single dose of 50 or 100 mg/kg of CCT271850. Tumours and plasma samples were collected at 2, 6, 12 and 24 h after CCT271850 dosing and followed with PK and PD examination. Samples were lysed and analysed by MSD for pTpS^33/37^ and GFP. Ratio of phospho-MPS1 (pTpS^33/37^)/Total-MPS1 (GFP) in tumour samples at various time points after 50 and 100 mg/kg CCT271850 dosing was calculated. Concentration of CCT271850 in tumours (**E**) and plasma (**F**) was measured.

The indirect readout of the inhibition of histone H3 phosphorylation at S10 has been widely used as a biomarker for inhibition of MPS1 (Colombo *et al*, 2010; Kwiatkowski *et al*, 2010; Santaguida *et al*, 2010; Tardif *et al*, 2011). Mitotic exit following MPS1 inhibition leads to a robust reduction in histone H3 phosphorylation. Similar modulation of other mitotic markers such as cyclin B levels has also been shown in response to MPS1 inhibition (Colombo *et al*, 2010; Tardif *et al*, 2011). Although direct inhibition of MPS1 autophosphorylation using phospho-specific antibodies has been reported in cells (Sliedrecht *et al*, 2010; Tardif *et al*, 2011), such inhibition has never been demonstrated in *ex vivo* tumour samples. In addition, although a direct inhibition of phosphorylation of the natural substrate of MPS1, KNL1, by MPS1 inhibitors has been described using immunohistochemistry (Maia *et al*, 2015), this method it is not as quantitative as an ELISA-type method such as MSD assay. To directly measure MPS1 inhibition using CCT271850 in a quantitative method, we have recently generated stable DLD1 cell line clones where GFP-MPS1 is overexpressed in a Dox-inducible manner (Gurden *et al*, 2015). To measure autophosphorylation of MPS1 *in vivo*, we subcutaneously injected DLD1-GFP-MPS1 cells into mice to establish xenograft tumours that express ectopic MPS1 upon induction by Dox. Despite the levels of GFP-MPS1 being only 1.5-fold higher compared to endogenous MPS1 (band intensity of GFP-MPS1 *vs* endogenous MPS1; Figure 4A), the signal for MPS1 autophosphorylation at T33/S37 in MSD assay was robustly increased in DOX-induced tumours, compared to the non-induced tumours (Figure 4B). The expression of GFP-MPS1 in these tumour samples was also confirmed by the MSD assay in a PK/PD study conducted with CCT271850 compound in the inducible DLD1 xenograft tumour (Figure 4C). Importantly, at 2, 6 and 12 h after a single dose of 100 mg/kg of CCT271850, there was >90% inhibition of MPS1 autophosphorylation, which dropped to 79% at 24 h (Figure 4D). When a single dose of 50 mg/kg of CCT271850 was used, the inhibition ranged from ∼89% at 2 and 6 h, to 61% at 24 h after treatment. At 12 h, 50 and 100 mg/kg dosing of inhibitor caused 90% and 78% reduction in MPS1 phosphorylation respectively. When analysing the tumour compound concentration, we found that concentration of CCT271850 in the tumours was >1 *μ*M at all time points, which is significantly higher than the IC_50_ value of CCT271850 in DLD1 cells (396 nM; Figure 4E). The plasma concentration of CCT271850 was comparable at 2 and 6 h time points but gradually decreased at 12 and 24 h (Figure 4F). The greatest drop in inhibition of MPS1 phosphorylation was observed for 50 mg/kg at 24 h, which correlated with the lowest levels of the compound in both plasma and tumour. Similar results were obtained when 100 mg/kg of CCT251455, our first published MPS1 inhibitor (Naud *et al*, 2013), were dosed in mice bearing Dox-induced DLD1 xenografts. Autophosphorylation of MPS1 at the T33/S37 sites was completely inhibited in tumours collected at 2 and 6 h after treatment, whereas the levels of MPS1 overexpression (GFP) measured by MSD assays were similar in all treated and control samples (Supplementary Figure S4).

### Efficacy of CCT271850 in HCT116 xenografts

**Figure 5:**
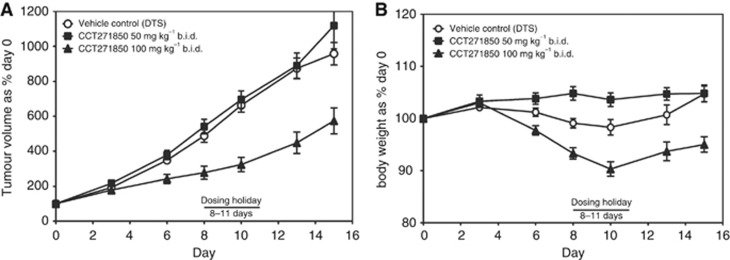
**Efficacy of CCT271850 in HCT116 xenografts.**Athymic mice bearing HCT116 tumours were dosed with vehicle, 50 or 100 mg/kg of CCT271850 orally twice daily (b.i.d) with a break after 1 week's dosing for days 0–7 and 12–15. (**A**) The mean tumour volumes±s.e.m. for control and CCT271850-treated mice were measured at different days throughout the experiment. (**B**) Body weights for treated and control mice were measured.

We then examined the efficacy of CCT271850 *in vivo* using HCT116 tumour xenografts. Mice bearing tumour xenografts were dosed twice daily with 50 or 100 mg/kg of CCT271850 for days 0–7 and 12–15. Moderate level of efficacy at the dose of 100 mg/kg was achieved with tumour *vs* control (T/C) of 60% based on the final tumour volume (Figure 5A). Treatment with CCT271850 was well tolerated in both rounds of dosing with less than 8% body weight loss compared with the vehicle-treated controls over 15 days (Figure 5B). While this T/C is still modest, it has been shown in the literature that only moderate efficacy can be achieved in mouse models with tolerated doses of MPS1 inhibitors as single agent in the models that have been tested (Colombo *et al*, 2010; Tardif *et al*, 2011; Laufer *et al*, 2014; Kusakabe *et al*, 2015; Maia *et al*, 2015), indicating that combination studies with standard-of-care may lead to a more successful use of MPS1 inhibitors in clinic in this particular type of cancer.

## Discussion

Mitosis has attracted a large number of studies focusing on cancer for decades – either to identify mitotic mechanisms involved in cancer, or at a translational level, to target fast proliferating cancer cells using antimitotic drugs. There is no doubt that antimitotics are a success in certain types of cancer, but the lack of differentiation between normal and cancer cells underlines their limitations in clinic. Mitotic kinases have been identified as essential proteins in regulating mitosis and therefore they have represented an attractive target in cancer therapy during the past two decades.

MPS1 is an essential kinase in initiating the SAC, which, in cooperation with Aurora-B kinase that predominantly maintains SAC signalling (Gurden *et al*, 2016), represents the cornerstone of this surveillance mechanism. MPS1 has been found to be deregulated in a variety of human cancers and has been associated with certain genetic abnormalities characterised in tumour cells, including chromosomal instability (Carter *et al*, 2006) and aneuploidy (Brough *et al*, 2011; Gordon *et al*, 2012). RNAi-mediated knockdown or chemical inhibition of MPS1 has also been shown to mediate cell death in PTEN-deficient breast tumour cells (Brough *et al*, 2011). Therefore, MPS1 has been brought to the attention as an anticancer target.

Here we present the discovery of CCT271850, a novel, oral, potent and selective MPS1 inhibitor in biochemical and cellular assays, with *in vivo* activity. CCT271850-induced growth inhibition was observed in a wide range of cancer cell lines. The highest sensitivity was observed in colon cancer cell lines, particularly the MSI-positive colon cancer cell lines in comparison to MSS. In addition, basal breast cancer cell lines, some of which are also PTEN-deficient, showed higher sensitivity to cell death upon MPS1 inhibition in comparison to luminal breast cancers, indicating a potential patient stratification for treatment with an MPS1 inhibitor. Consistent with MPS1 inhibition, CCT271850-treated cells lost kinetochore localisation of the checkpoint proteins BUBR1, MAD1 and MAD2, spent less time in mitosis compared to untreated cells, underwent an abnormal division with unaligned chromosomes, which caused aneuploidy and cell death. We have previously shown that following MPS1 inhibition, the majority of the cells die after one or two mitoses (Gurden *et al*, 2016), indicating a distinct difference between MPS1 inhibitors in comparison to other mitotic kinase inhibitors such as Aurora and Polo-like kinase inhibitors; these inhibitors require multiple cell cycles to induce cell killing, and thus have a narrower therapeutic window in clinic (Bavetsias, Linardopoulos, 2015). These effects of CCT2571850 were specifically caused through MPS1 since cell lines, previously generated (Gurden *et al*, 2015), containing a S611G mutation in MPS1 were resistant to inhibition.

The lack of a direct, quantitative biomarker for MPS1 inhibition in order to identify a PD *vs* efficacy relationship prompted us to set up and optimise an inducible MPS1 model in DLD1 colon cancer cells, in order to directly measure MPS1 inhibition via MPS1 autophosphorylation. Assays using MSD have been proved to be very sensitive and quantitative *in vitro* and *in vivo*. We validated and characterised the specificity of a commercially available T33/S37 anti-MPS1 phospho-antibody, which we used to analyse MPS1 activity in biochemical assays, in cell and *in vivo*. We showed that 100 mg/kg of CCT271850 is sufficient to inhibit MPS1, *in vivo,* for at least 24 h in HCT116 human tumour xenografts, by ∼80%, as shown by a decrease in MPS1 autophosphorylation levels. This level of inhibition resulted in a moderate effect on tumour growth, suggesting that higher, more sustained target inhibition may be required for a more robust tumour response. Likewise, although 50 mg/kg showed robust target inhibition for up to 12 h, this was insufficient to produce any reduction in tumour volume. However, since higher dosing regiments are limited by animal toxicity, a single agent administration may not be as efficacious as is required in clinical settings in the type of cancer models tested. This suggests that changing direction towards using MPS1 inhibitors in combination with standard-of-care drugs, for example, with taxanes, may be a more beneficial approach for the clinic. It has been recently shown that paclitaxel induces multipolar spindles and mitotic delay (Zasadil *et al*, 2014). Our hypothesis is that MPS1 inhibition will induce abrogation of paclitaxel-induced mitotic delay causing gross chromosomal abnormalities and massive cell death in cancer cells.

## Figures and Tables

**Table 1:** IC_50_ of CCT271850 in *in vitro* and cell-based assays

	**CCT271850 (μM)**
MPS1 IC_50_: Caliper 10 *μ*M ATP	0.011±0.004
MPS1 IC_50_: Caliper 1 mM ATP	0.02±0.013
MSD HCT116 IC_50_	0.059±0.022
P-histone H3 IC_50_	0.067±0.004
3 Days MTT HCT116 IC_50_	0.151±0.006

Abbreviations: IC_50_=half maximal inhibitory concentration; MSD=meso scale discovery; MTT=3-(4,5-dimethylthiazol-2-Yl)-2,5-diphenyltetrazolium bromide.

**Table 2:** IC_50_ of CCT271850 determined by MTT and clonogenic assays (CA)

**Cell line**	**MSI/MSS**	**3-Day MTT (μM)**	**14-Day CA (μM)**
SW620	MSS	0.065	0.02
HCT-15	MSI	0.102	0.042
HRT-18	MSI	0.116	0.024
HCT116	MSI	0.149	0.0226
SW48	MSS	0.171	0.037
MAWI	ND	0.172	0.051
SW948	MSS	0.18	0.013
COLO205	MSS	0.284	0.016
PC/JW2	ND	0.286	0.036
LS174T	MSI	0.306	0.037
RKO	MSI	0.341	0.06
DLD1	MSI	0.396	0.041
LOVO	MSI	0.526	0.054
T84	MSS	0.617	0.015
WiDr	MSS	0.82	0.02
SW403	MSS	1.031	0.009
HT-29	MSS	1.124	0.07
COLO741	MSS	1.542	0.022
COLO320	MSS	1.752	0.078
SW480	MSS	1.838	0.044
SW1417	MSS	2.798	0.107
HT55	MSS	2.923	0.024
SW837	MSS	3.258	0.07
SNU-C2B	MSI	>100	0.059

**Cell line**	**Subtype**	**PTEN**	**3-Day MTT (μM)**	**5-Day MTT (μM)**	**14-Day CA (μM)**
CAL51	Basal B	Null	0.068	0.044	0.015
SUM159PT	Basal B	WT	2.075	0.081	0.046
BT474	Luminal A	WT	0.123	0.09	0.039
SUM149PT	Basal B	Null	0.51	0.148	0.052
MDA-MB-468	Basal A	Null	0.82	0.24	0.027
BT549	Basal B	Null	1.483	0.407	0.174
EFM-19	Luminal	WT	1.23	0.473	0.044
HS578T	Basal B	WT	8.772	0.832	0.042
HCC1143	Basal A	WT	2.59	0.957	0.093
MDA-MB-157	Basal B	WT	5.392	1.131	0.059
MCF7	Luminal B	WT	2.377	1.146	0.011
KPL-1	Luminal	WT	2.215	1.266	ND
BT20	Basal A	WT	2.67	1.613	0.037
MDA-MB-231	Basal B	WT	7.945	1.706	0.053
ZR75.1	Luminal B	Null	2.999	1.733	0.04
HCC1428	Luminal B	WT	1.03	1.784	ND
T47D	Luminal B	WT	2.02	2.183	0.065
MDA-MB-361	Luminal	WT	0.705	3.238	ND
CAMA1	Luminal	WT	7.157	4.01	0.036
MDA-MB-453	Luminal B	WT	2.21	5.67	ND

Abbreviations: MSI=microsatellite instability; ND=not determined; WT=wild type.

All cell lines were tested at least two times.
