# Supplementary figures and images for: Characterisation of CCT271850, a selective, oral and potent MPS1 inhibitor, used to directly measure in vivo MPS1 inhibition vs therapeutic efficacy
Source: Br J Cancer. 2017 Mar 23;116(9):1166–76. doi: 10.1038/bjc.2017.75 (PMC5418449; doi:10.1038/bjc.2017.75)

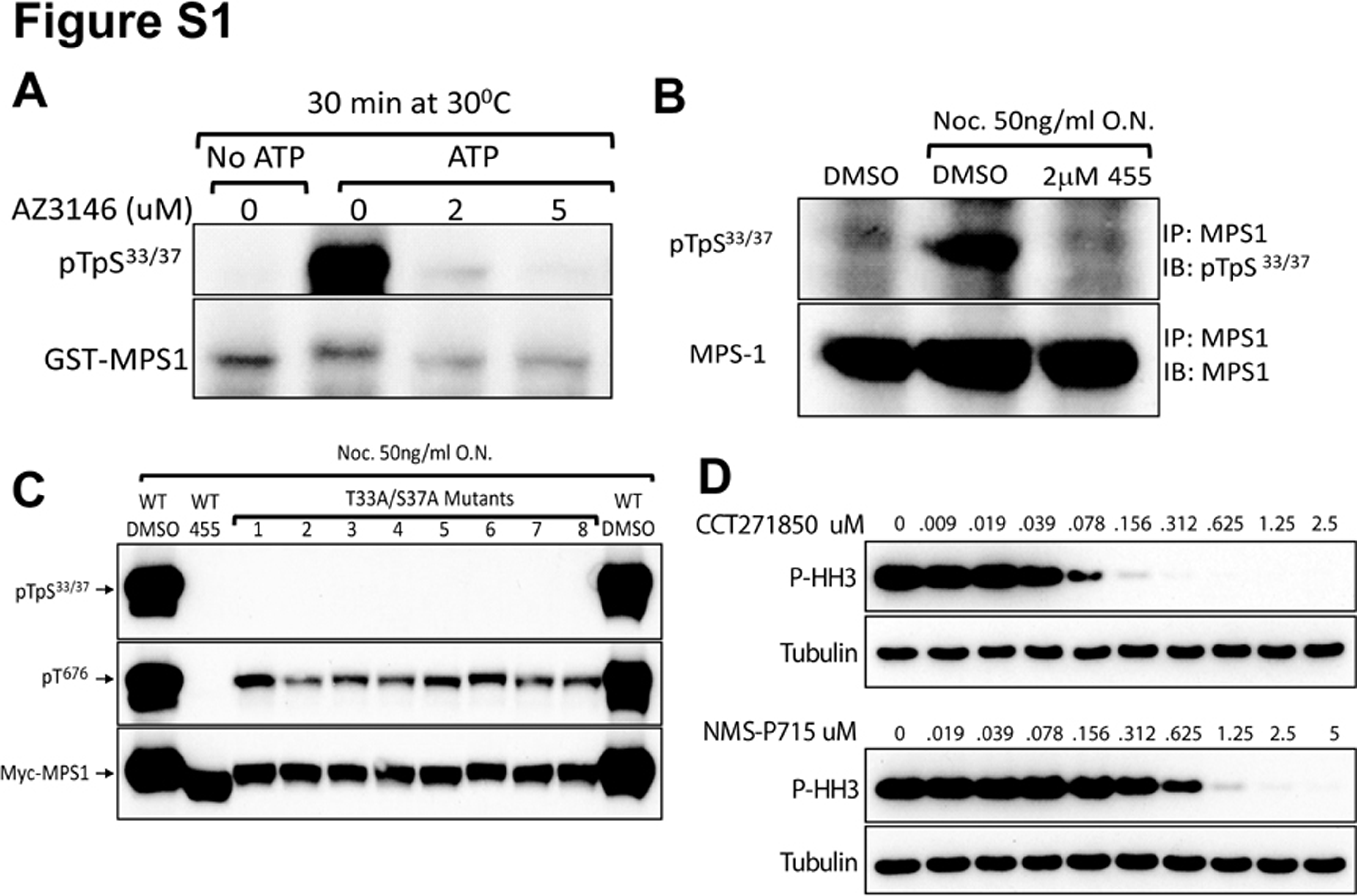

Supplement: Supplementary Figure S1 [file bjc201775x1.tif]

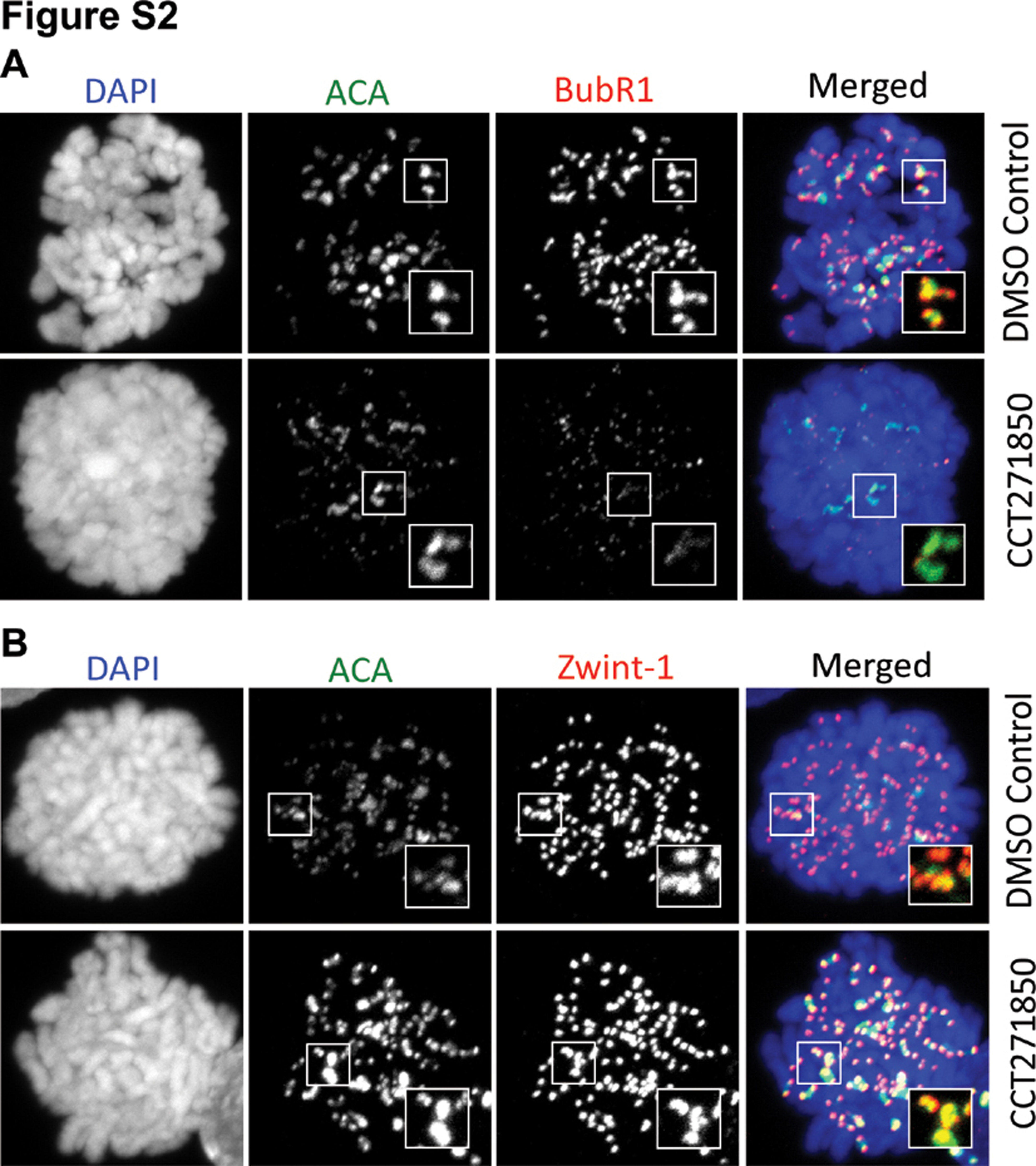

Supplement: Supplementary Figure S2 [file bjc201775x2.tif]

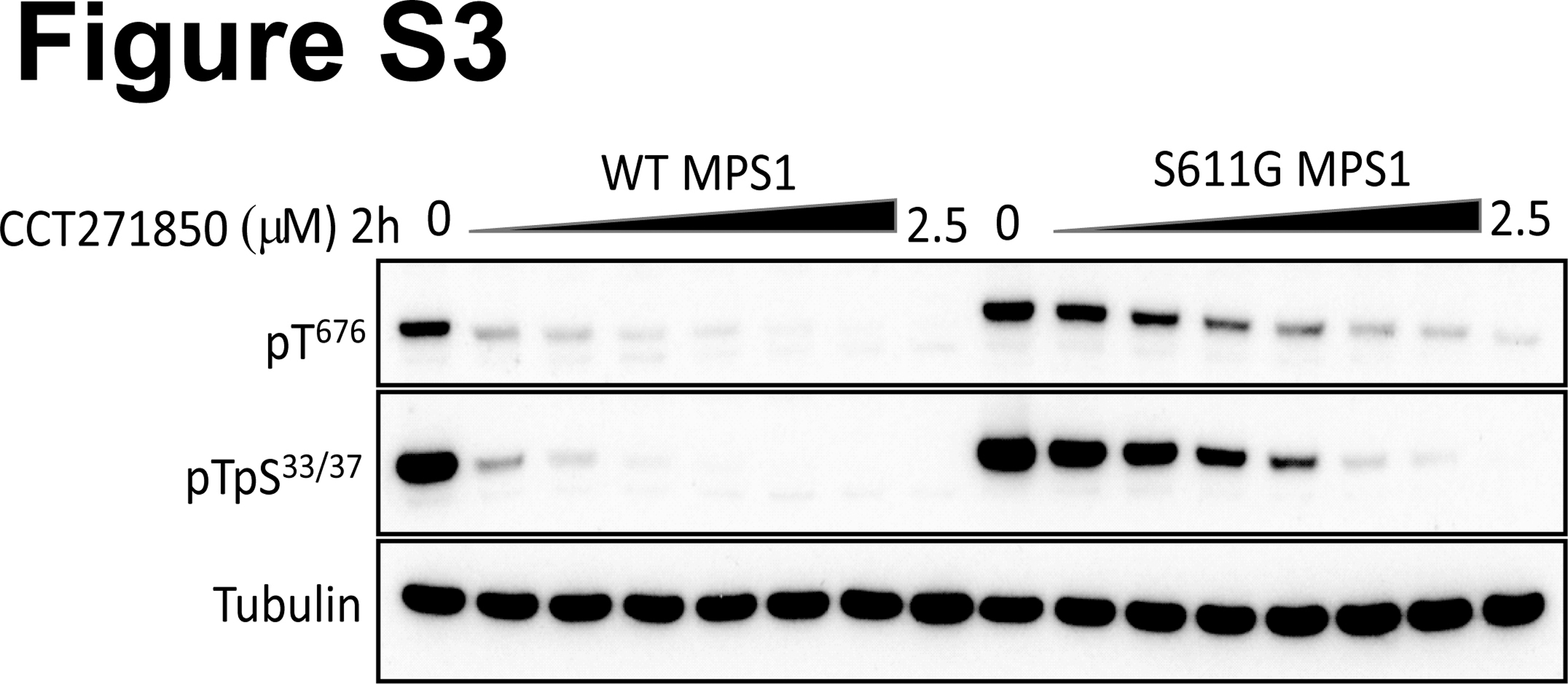

Supplement: Supplementary Figure S3 [file bjc201775x3.tif]

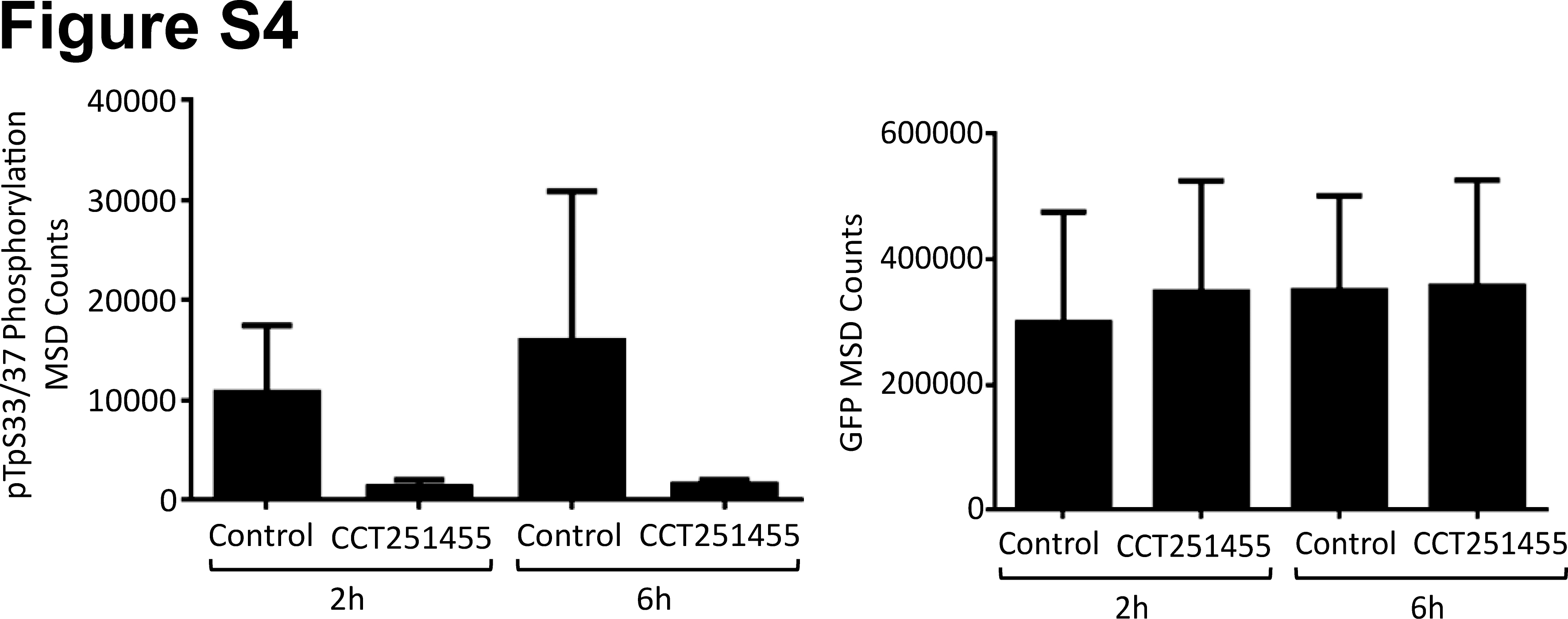

Supplement: Supplementary Figure S4 [file bjc201775x4.tif]
